# Development of a Conductivity Sensor for Monitoring Groundwater Resources to Optimize Water Management in Smart City Environments

**DOI:** 10.3390/s150920990

**Published:** 2015-08-26

**Authors:** Lorena Parra, Sandra Sendra, Jaime Lloret, Ignacio Bosch

**Affiliations:** 1 Integrated Management Coastal Research Institute, Universitat Politècnica de València, C/Paranimf, n° 1, 46730 Grao de Gandia, Spain; E-Mails: loparbo@doctor.upv.es (L.P.); sansenco@posgrado.upv.es (S.S.); 2 Institute of Telecommunications and Multimedia Applications (iTEAM), Universitat Politècnica de València, Camino Vera n/n, 46022 Valencia, Spain; E-Mail: igbosroi@dcom.upv.es

**Keywords:** conductivity sensor, groundwater monitoring, water management, Smart City, saline intrusion, solenoid coils

## Abstract

The main aim of smart cities is to achieve the sustainable use of resources. In order to make the correct use of resources, an accurate monitoring and management is needed. In some places, like underground aquifers, access for monitoring can be difficult, therefore the use of sensors can be a good solution. Groundwater is very important as a water resource. Just in the USA, aquifers represent the water source for 50% of the population. However, aquifers are endangered due to the contamination. One of the most important parameters to monitor in groundwater is the salinity, as high salinity levels indicate groundwater salinization. In this paper, we present a specific sensor for monitoring groundwater salinization. The sensor is able to measure the electric conductivity of water, which is directly related to the water salinization. The sensor, which is composed of two copper coils, measures the magnetic field alterations due to the presence of electric charges in the water. Different salinities of the water generate different alterations. Our sensor has undergone several tests in order to obtain a conductivity sensor with enough accuracy. First, several prototypes are tested and are compared with the purpose of choosing the best combination of coils. After the best prototype was selected, it was calibrated using up to 30 different samples. Our conductivity sensor presents an operational range from 0.585 mS/cm to 73.8 mS/cm, which is wide enough to cover the typical range of water salinities. With this work, we have demonstrated that it is feasible to measure water conductivity using solenoid coils and that this is a low cost application for groundwater monitoring.

## 1. Introduction

The Smart City concept covers several aspects. It is based on the integration of Information and Communications Technology (ICT) systems in urban environments to improve the use of resources and reduce the emissions of harmful gases in order to achieve the sustainability of cities [1,2]. It is possible to find some papers where authors work on the concept of sustainability for smart cities. Some examples such as [3,4] are focused on saving energy and public transport for improving the heat and energy management. There are even some works where the authors present a smart grid for water distribution in a smart city [5]. However the most common applications are light control methods for smart grids, as can be seen in [6,7,8,9]. To obtain drinkable water and how it is distributed in cities is not addressed in any research work for smart cities, although it is one of the most important resources in cities [10]. Water monitoring, mainly in natural environments, is a hot topic and it is discussed in several papers such as the one presented in [11]. Climate change, the increase of population and the increase of water pollution are making this challenge bigger.

### Problem Formulation

In this subsection, we are going to analyze the problem of sustainability of groundwater resources and how different factors affect it. The worldwide population is increasing. Several studies have modeled this growth and made predictions. According to United Nations data [12], in 2010, the world population was near 7 billion people. This value will increase up to 9.3 billion in 2050 and will reach 10 billion in 2100. One of the most important resources demanded by the population is fresh water. However, the amount of fresh water in the world is limited. Water is a recirculating resource and it must be properly managed to ensure its continued availability. According to [13], 2.5% of the total water in the world is fresh water, 0.75% of the total water is stored in groundwater and only the 0.0072% of water is contained in lakes, rivers and swamps. The groundwater volume is 100 times bigger than the volume of water in river and lakes, however it is little used. Groundwater supplies suppose more than 25% by volume of the total supply of water in USA (with an estimated value of 20.09 billion dollars) [14,15]. In some rural areas, groundwater is the only water source and it is used for agriculture and for households. The water contained in aquifers supplies up to 50% of the US population [16]. While groundwater seems to be an important supply in many areas, its contamination problems must be prevented by applying good management practices to avoid major problems. Particularly, groundwater salinization is the major groundwater contamination issue in the world [17].

Groundwater contamination is generally linked to runoff and infiltration of surface water from urban and agricultural areas. The salinization problem is related to coastal zones where the aquifer arrives near the sea. Sometimes it is very difficult to avoid mixing freshwater with seawater. This is because freshwater emerges in some marine areas, but if too much water is extracted from the aquifer, then, seawater can filtrate into the aquifer. If this happens, the groundwater will not be drinkable for a long time. In addition, as we can see in some simulations [18], climate change also influences the aggravation of this effect. The urbanization process also affects to the natural equilibrium due to the soil waterproofing that reduces the local infiltration (however, according to some authors, the global infiltration increases [19]). In [20] authors demonstrate that urbanization process has effects in groundwater and that the effect can continue through the time even when the process has finished. According to Padowski *et al.* (stated in their work published in 2010 [21]) among the 70 largest cities in the world, 28 of them (40%) use only groundwater supplies. Of these 28 cities only six of them use a non-threatened reservoir, 20 depend on a threatened reservoir and two on a vulnerable one. However, simulations for 2040 show us that only three of these cities will have their reservoirs not still threatened and five cities will have vulnerable reservoirs. This situation can be extrapolated to the rest of the world. The aquifers are endangered and those aquifers are the water source of many people.

**Figure 1:**
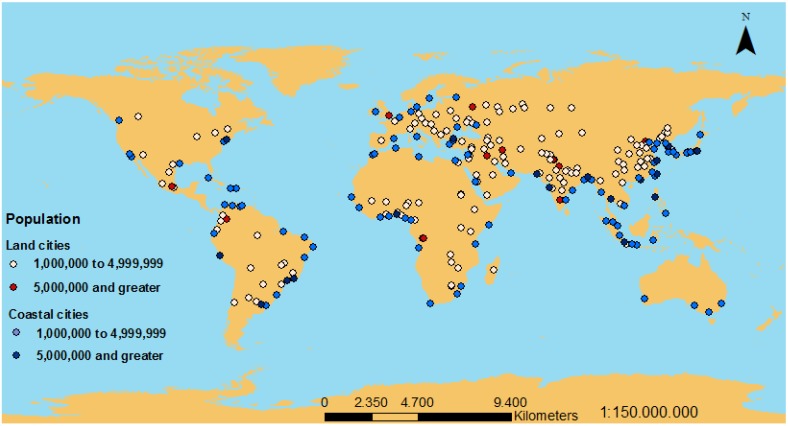
Distribution of cities with more than 1 Million of inhabitants.

In the last decades, the population is increasing and the migration effect is making that population concentrate in coastal areas, so cities in coastal areas have suffered a dramatically growth in very few years. Using data of the cities with more than 1 million population [22], we calculated the percentage of cities that can be considered as coastal cities (50 km or nearer from the sea). The distribution of cities with more than 1 million inhabitants is shown in Figure 1 and these cities are represented in different colors depending on their proximity to the sea. Table 1 shows the number of cities considered as coastal or inland. It can be seen that 60% of large cities are placed at less than 50 km from a coast.

**Table 1:** Percentage of cities with more than 1 M (Million) of inhabitants considered as coastal.

Cities Population	Coastal	Inland	% Coastal
More than 5 M	18	30	60
Between 1 M and 5 M	87	226	38
Total population of these cities	358,350,495	719,607,161	50

In summary, groundwater is the water supply for a great part of the world population. The major part of the world population is living in coastal zones. In those areas the groundwater is susceptible to saline intrusion which can make the water undrinkable. The urbanization process, the climate change and the pumping of groundwater can alter the coastal aquifer equilibrium between salt and fresh water. All these facts lead to an alarming situation for the sustainability of cities and some researchers already mentioned and rationalized the necessity of groundwater monitoring [20,23] while others are making some measurements and simulations. However, the simulations and extrapolations are not so accurate in this case due to the high number of variables that affect each aquifer. Each city must be considered as a specific case requiring specific monitoring and diagnostic [23]. In order to monitor the groundwater parameters, and specifically the salinity, current efforts are based on manual sampling of wells with different time periodicity. This methodology supposes a waste of energy, money and human efforts compared with the possibility of using wireless sensor networks (WSNs) [24,25]. The use of WSNs for conductivity monitoring makes possible the real-time collection of data and high spatial resolution (if we need to monitor a high number of accessible wells) and it is a good option for long-term monitoring.

In this paper, we present a specific conductivity sensor designed for monitoring groundwater. The sensor is based on solenoid coils and covers all the requirements for groundwater environments. It can be easily isolated from the environment with different materials. It does not need periodic calibration and has low energy consumption, so it can be left in groundwater for long term monitoring. The operational range of the sensor fits perfectly with groundwater monitoring requirements. The sensor is robust and easy to clean (if necessary). Moreover, there are no papers where different prototypes of solenoid coils are tested in different conditions to evaluate their potential as a salinity sensor.

The rest of the paper is structured as follows: Section 2 presents some previous works about sensors for groundwater, current conductivity studies in groundwater and conductivity sensors and some background theory. Section 3 explains the composition of our conductivity sensor and the test bench performed to test its operation. The obtained results are presented in Section 4. Finally Section 5 contains the conclusions and the future work.

## 2. Related Work

This section is divided into several subsections in order to provide a general overview of the different areas that cover the topics of this paper. First, in Section 2.1, we review published conductivity studies in groundwater. Section 2.2 shows a summary of works where authors present the conductivity status or changes in groundwater. This subsection also analyzes the need of using a conductivity sensor for conductivity monitoring in groundwater. Finally, Section 2.3 presents the state of art of conductivity sensors.

### 2.1. Conductivity Studies in Groundwater

This subsection shows a compilation of the main studies carried out by other authors about the conductivity status and its evolution in groundwater all over the world. Table 2 presents several examples where authors studied the value of conductivity in groundwater. To study the salinization process of groundwater, authors analyzed the value of Electrical Conductivity (EC) or Total Dissolved Solids (TDS) among others. EC and TDS are water properties that bring general information about the salinization level. Authors studied the % of different ions or even the presence of specific isotopes to obtain more detailed information. Nevertheless, the values of EC or TDS themselves are good indicators of changes. Besides, they are easier to measure than the presence of a specific ion. Some studies analyzed TDS [26,27,28,29], others studied the EC [30,31,32,33,34,35,36], a small part of them studied both parameters [37,38,39] and, in [40], other authors measured other parameters (g/L). Most of these studies (60%) analyzed the problem of salinization from a static point of view. On the other hand, 40% of the performed studies are based on dynamic data analysis collected during different years. However, not all of these studies analyzed the same phenomena. Works presented in [27,28] were focused on long term evolution but authors studied the anthropogenic and natural causes and do not focus the work on the extraction of water supplies.

Only three of the previous papers presented in this related work evaluated the changes of salinity in groundwater with water pumping, agricultural pumping in [38,40] and industrial pumping in [34]. However, we have observed that there are no papers providing long-term measurements in urban environments. For that reason, in this paper a specific conductivity sensor for groundwater conductivity monitoring is presented.

**Table 2:** Summary of current studies of salinization process in groundwater.

Ref.	Type of Study	Sampling Period	Number of Wells	Study Area (km^2^)	Country	EC/TDS	Conductivity Range (mS/cm)	Publish Year
[26]	Static	1 Sampling period (2000/2001)	n/a	500,000	Korea	TDS	n/a	2005
[37]	Static	1 Sampling period (2001)	18	1845	Korea	Both	0.114 to 25	2003
[30]	Static	1 Sampling period (2009)	79	35	Italy	EC	0.795 to 4.72	2012
[31]	Static	1 Sampling period (2006)	41	190	Morocco	EC	2.55 to 21	2009
[32]	Static	3 Sampling period (2005/2006)	8	750	France	EC	0.1 to 57.9	2008
[33]	Static	1 Sampling period (2006)	n/a	n/a	Greece	EC	0.5 to 24	2009
[29]	Static	1 Sampling period (2001)	n/a	n/a	China	TDS	n/a	2005
[35]	Static	5 Sampling period (2007)	55	n/a	Turkey	EC	0.1 to 42.8	2011
[36]	Static	1 Sampling period (2000/2001)	69	n/a	Australia	EC	12.7 to 17.3	2006
[38]	Dynamic	1968 to 1995	35	1900	Mexico	Both	0.878 to 4.910	2004
[34]	Dynamic	1984 to2000	4	n/a	Turkey	EC	0.807 to 0.924	2004
[27]	Dynamic	1994 to 2004	26	1200	Italy	TDS	n/a	2011
[28]	Dynamic	1960 to 2010	n/a	90,000	U.S.A	TDS	n/a	2014
[40]	Dynamic	1996 to 2005	24	16,100	Uzbekistan	Other	n/a	2009
[39]	Dynamic	1999 to 2001	40	n/a	India	Both	2.4 to 2.6	2008

n/a = Information not available.

### 2.2. Salinity Sensors

In this subsection a review of the main conductivity sensors is presented. First, the different methods for measuring the conductivity in fluids are discussed. A comparative table is also presented where different conductivity sensors with different operational principles are compared.

There are different methods to measure the salinity of a liquid based on the changes of physical parameters between freshwater and saltwater. Those parameters are: density, light refraction and electrical conductivity (EC) [41,42]. However, due to its application, changes in density are not as useful as other parameters. Firstly, we analyze the use of light refraction to measure the salinity of water. When salinity of a liquid increases, the refraction angle of an incident light diverges. The saltier the water is, the more divergent the light incident angle is [42]. Different sensors have been developed based on this principle [42,43,44]. The sensors present good resolution and accuracy. However, their use in long term monitoring can result complicated. The need of cleaning surfaces where the light is in touch of samples is a big problem in water environments. Different bacteria and sediments can precipitate on the surface producing errors in the measurements inducing wrong results.

The last option is to measure the electric conductivity (EC) of the liquid; higher salinity entails higher electric conductivity. The previous subsection showed how several authors used the EC to evaluate the salinity of groundwater samples. There are two different methods to measure the EC, the conductive methodology and the inductive methodology. The first one is based on the transmission of electric current through the water. It uses two copper electrodes, the first one is powered with an electric current and the second electrode is placed near to the first one. The second one receives part of the electric current. The amount of current that receives the second electrode depends on the electric current of the first electrode, area of electrodes, distance between electrodes and water salinity. The electrodes must be in contact with the water. This supposes some drawbacks such as corrosion and the deposition of fine material or bacteria that can produce alterations on the transmission of electric current from the powered electrode. For long term monitoring, the electrodes must periodically be cleaned and replaced. That does not fit with the aim of having a low cost WSN for long term monitoring.

The inductive methodology is based on the attenuation of an electromagnetic field in the liquid. It uses two copper coils. One of them is powered and generates an electromagnetic field, and the second one presents an induced current due to that electromagnetic field. The magnitude of the induced current depends on several factors. However those factors are not so studied as in the case of conductive methodology. Generally, the size of coils [45], the salinity of water [45,46,47,48] and even the volume of water [49] are defined in different related work as important factors. This methodology makes it possible to isolate the copper parts from the water, as exposed in [48]. Despite the great benefits this system offers, it is not widely used. The first time researchers mentioned measuring salinity using magnetic fields was in 1985 [50]. Since then, few works have used this method. [45,46] are the sole works found where inductive methods are used. In these cases, the authors only used two coreless toroid coils to perform these kinds of measurements. In 2013, our research group started to perform a set of experiments using different coil combinations [48], and combinations of coils and Hall sensors [47] and to study the effect of water volume [49]. Our previous conclusions suggested that the use of solenoid coils was as valid as the use of toroid coils. Only in [48] the authors evaluated the induced voltage at different frequencies with different prototypes.

With the aim of creating a specific and simply sensor for groundwater monitoring, in this paper, we continue our test with two solenoid coils. Table 3 shows a summary of all the papers where the authors used conductivity sensors based on both methodologies. In this table, we compare the current sensors for conductivity monitoring. As we can see, only the inductive ones are able to operate in a measurable range that matches with the needs of groundwater monitoring from 1 mS/cm (or even less) to 57.9 mS/cm.

**Table 3:** Summary of current sensors for electric current monitoring.

Methodology	Sensor Description		Measurable Range (mS/cm)	Match with Measurable Needs	Publication Year	Ref.
Inductive	2 Toroid	Same diameter	1 to 44	Possible	2006	[46]
		Same diameter (1.125 inch)	3 to 48	No (To High)	2010	[45]
		Same diameter (2.125 inch)	0.45 to 3.4	No (To Low)		
		Different diameter	0.397 to 90.3	Good	2013	[48]
	Other	**2 Solenoids**	**0.397 to 90.2**	**Good**		
		1 Toroid and 1 Solenoid	0.397 to 90.4	Good		
		1 Solenoid + sensor Hall	0.0028 to 194	Good	2013	[47]
		1 Toroid and 1 Solenoid	0.397 to 76	Good	2013	[49]
Conductive	H-bridge and digital potentiometer		15.6 to 53.9	No (To High)	2010	[51]
	4 Electrodes in a pipe		0.5 to 6.5	No (To Low)	2008	[52]
	Interdigitate electrodes	4 Electrodes in a pipe	0.007 to 0.32	No (To Low)	2002	[53]
		4 electrodes	0.33 to 14.64	No (To Low)	2013	[54]
		7 electrodes	25 to 55	No (To High)	2011	[55]
		4 models (40 and 63 electrodes)	32 to 60	No (To High)	2011	[56]
		Several electrodes	0.12 to 12	No (To Low)	2014	[57]

### 2.3. Background Theory

**Figure 2:**
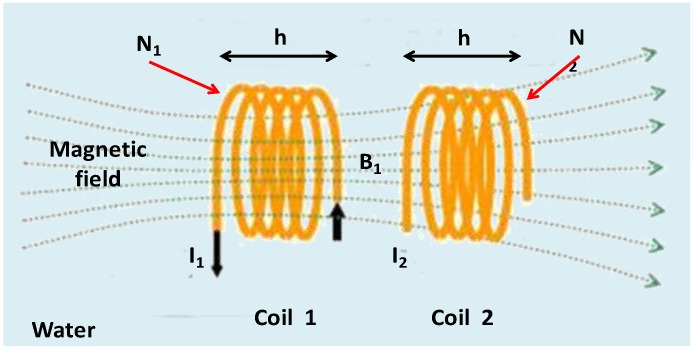
Electric circuit of the sensor.

To understand the operation of our sensor, we need to understand the concept of mutual inductance. We are going to explain the how it works by using the scenario shown in Figure 2. We have two coils with a length h, where $N_{1}\;$ and $N_{2}\;$ are the number of turns of each coil, respectively. Instead of having a ferromagnetic core with a section S  and a relative permeability $\mu_{r}$. We have a space occupied by salt water where the section of the coils is $N_{1}\;$ and the relative permeability $\mu_{r\_water}$.

Throughout the coil 1 a constant current ${\text{I}}_{1}$ flows, while the coil 2 is open. To simplify the equations system, let us assume that all lines of the magnetic field created by the coil 1 flow through the coil 2. Coil 1 creates a magnetic field ${\vec{B}}_{1}$. This magnetic field is confined to the center of the coil 1, as if it were a core. In this way, the lines of the magnetic field ${\vec{B}}_{1}$ go through the coil 2 and create a magnetic flux $\Phi_{2,1}$. The mutual inductance is shown in Equation (1):

(1) $$L_{1,2}=M=\frac{\Phi_{2,1}}{I_{1}}$$

On the other hand, the magnetic field in coil 1 is given by:

(2) $${\vec{B}}_{1}=\mu_{r\_water}\cdot \;\mu_{0}\frac{N_{1}}{h}\cdot I_{1}\cdot \vec{n}$$

where $\vec{n}$ is unitary vector, parallel to the axis coils and it is directed to the right side. The flow produced on the coil 2, $\Phi_{2,1}$, is shown in Equation (3):

(3) $$\Phi_{2,1}=N_{2}\cdot {\vec{B}}_{1}\cdot S=\frac{\mu_{r\_water}\cdot \;\mu_{0}\cdot \;N_{2}\cdot B_{1}\cdot S}{h}\cdot I_{1}$$

Equation (4) shows the coefficient of mutual inductance:

(4) $${\text{L}}_{1,2}=\text{M}=\frac{\mu_{r\_water}\cdot \;\mu_{0}\cdot \;{\text{N}}_{2}\cdot {\text{N}}_{1}\cdot \text{S}}{\text{h}}$$

On the other hand, the electromotive force in coil 2 ($emf{\;}_{2}$) can be calculated from the magnetic flow produced on the coil 2 which is given by Equation (5):

(5) $$\Phi_{2,1}=M\cdot I_{1}$$

If $I_{1}$ depends on the time, this flow also changes as a function of the time and generates an ${\text{emf}}_{2}$ which is given by Equation (6):

(6) emf2=−dΦ2,1dt=−M·dI1dt=Mτ·I0·e−tτ=μr_water· μ0· N2·B1·Sh·τ·I1·e−tτ

where τ is related with the working frequency of the induced ${\text{emf}}_{2}$.

Finally, we can conclude that the ${\text{emf}}_{2}$ is related with the medium through the variable $\mu_{r\_water}$ which is related with the amount of salts dissolved in the water.

## 3. Test Bench

In this section, we explain the components used in the developed tests. We also present the methodology carried out to analyze the dependence between the coil’s characteristics and its capability as a salinity sensor.

### 3.1. Methodology

As explained before, there are no studies that accurately evaluate the capability of coils to measure the salinity level of water. To study the different capability of each prototype, we have performed different tests. In each test, the prototypes are used to measure different saline solutions. We decided to study the effects of different parameters such as the number of windings (test benches 1 to 3), the diameter of the coils (test bench 4) and the effects of different copper wire diameter (test bench 5). Finally, we also studied the performance of each sensor working at different frequencies. Once the best configuration is selected, an exhaustive calibration is performed. The effect of water volume is also evaluated in order to determine the minimum cell volume [49].

### 3.2. Electric Circuit

**Figure 3:**
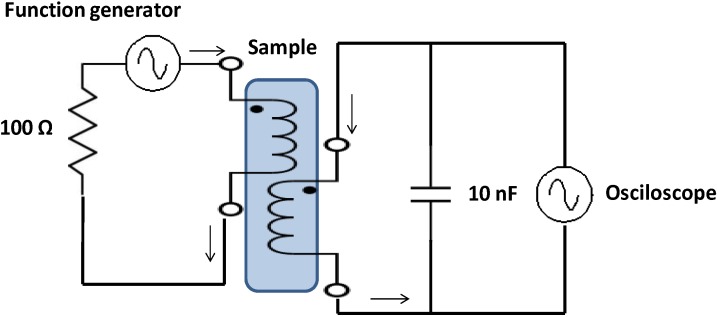
Electric circuit of the sensor.

The methodology used to measure the salinity is based on two electric solenoid coils, one of them is powered by a sinusoidal signal and the second one is connected to an oscilloscope in order to measure the induced magnetic field. This election is based on our previous works where we studied the best combination of coils [47,48,49]. Our results showed that the combination of two coils presented the lowest working frequency where a wide range of salinities could be differentiated. The electric coils are submerged in the water sample. The electric circuit is shown in Figure 3. The oscilloscope used is a HM303 and the function generator is an HP 33120A. The signal used to power the coils is a sinusoidal signal with a peak to peak voltage (*Vpp*) of 8 V and the used frequencies range from 100 kHz to 4000 kHz.

### 3.3. Electric Coils

The solenoids were coiled over PVC pipes of different diameters (depending on the test). The pipes have diameters of 15, 25 and 35 mm. The wire used to form the coils is enamel copper wire. Different diameters of wire are used in different tests. The diameters used are 0.4, 0.6 and 0.8 mm. The prepared solenoids had different number of windings where the smallest one has five windings and the biggest one has 120. The characteristics of all solenoids are shown in Table 4. Prototypes 5 to 9 were used in two tests using different setups and changing the powered and induced coils so appear twice, one in test 2 and other in test 3. When these prototypes are used in test 2, they are called as 5 to 9 and when these prototypes are used in test 3, prototypes are called as 5′ to 9′ in order to avoid confusions.

**Table 4:** Features of solenoids used in the tests.

Test	Prototype	Diameter of Wire (mm)	Diameter of Coils (mm)	Number of Windings of Powered Coil	Number of Windings Induced Coil	Windings Ratio
**1**	1	0.4	25	5	10	1:2
	2	0.4	25	10	20	1:2
	3	0.4	25	20	40	1:2
	4	0.4	25	40	80	1:2
**2**	5	0.4	25	30	15	1:0.5
	6	0.4	25	30	30	1:1
	7	0.4	25	30	60	1:2
	8	0.4	25	30	90	1:3
	9	0.4	25	30	120	1:4
**3**	5'	0.4	25	15	30	1:2
	6'	0.4	25	30	30	1:1
	7'	0.4	25	60	30	1:0.5
	8'	0.4	25	90	30	1:0.3
	9'	0.4	25	120	30	1:0.25
**4**	3	0.4	25	20	40	1:2
	10	0.6	25	20	40	1:2
	11	0.8	25	20	40	1:2
**5**	12	0.4	15	40	20	1:2
	3	0.4	25	20	40	1:2
	13	0.4	35	40	20	1:2

### 3.4. Preparation of Samples

In order to perform our tests, we need to prepare several samples. In our case, four different samples have been used. These samples have been prepared using tap water and adding different amount of salt. The conductivity of each solution is measured using a commercial device (CM 35 + conductivity meter). The salty solutions have different salinity levels, *i.e.*, the lowest one has 6.8 mS/cm and the highest one has 90.2 mS/cm. The typical sea water salinity is 52 mS/cm. One of our samples presents salinity close to that of sea water. The solutions were prepared at 7 °C.

On the other hand, we have prepared 30 samples (with salinities ranging between 0.585 mS/cm and 109.5 mS/cm) in order to perform the sensor calibration. The typical salinities registered in the groundwater with low and high levels of saline intrusion were included in this range of values.

All samples are prepared in round glass containers of 72 mm diameter and 150 mm height. All the containers had the same amount of water to be able to compare the measurements. Containers used in the test to evaluate the minimum cell volume, are also round glass containers. Their diameters are (in cm): 6.3, 7, 8 and 11.7. The biggest container is the one used for determining the minimum cell volume.

## 4. Results and Discursion

In this section, the test bench results are shown. This section is divided into three subsections in order to better show the obtained results. The first subsection corresponds to the test bench carried out for the salinity sensor characterization, where different configurations are tested. The second subsection shows the test bench results performed to know the minimum cell volume. Finally, subsection three presents the calibration of our sensor.

### 4.1. Physical Characterization of the Sensor

**Figure 4:**
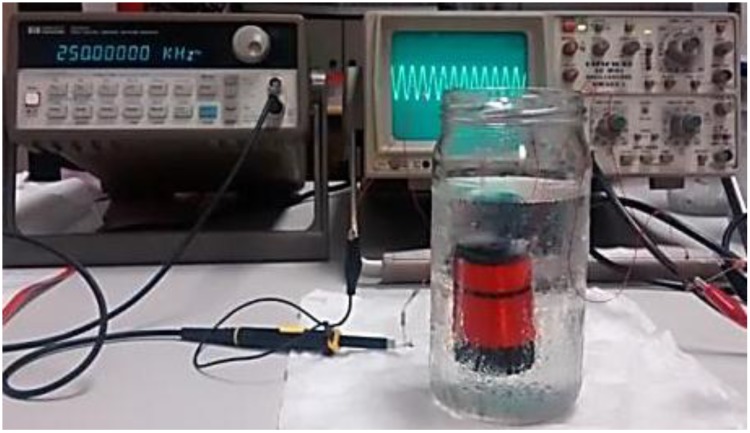
Picture of the test bench for one of the measurements.

In this subsection, we are going to present the results of the five tests performed to study the accuracy of the measurements when the parameters of coils are changed. Figure 4 shows the measurement process.

- Firstly, we study the effect of changing the number of windings but maintaining the windings ratio between the induced and the powered coil. For this test bench, we used prototypes from 1 to 4.
- The second test studies the change produced in the sensor performance when the windings ratio changes but maintaining the number of windings in the powered coil. For this test, we used prototypes from 5 to 9.
- The third test changes the number of windings of the powered coil while maintaining the number of windings of the induced coil. For this test, we used again prototypes from 5 to 9 but changing the powered coil by the induced coil from the second test.
- Fourth test bench evaluates the effect of changing the diameter of copper wire using three different diameters of copper wire while keeping equal the rest of parameters (number of windings in the coils and coil diameter). Prototypes 3, 10 and 11 were used in this test.
- Finally, a fifth test bench is performed to check the effect of changing the coil diameter but maintaining the rest of parameters (wire diameter and number of windings). Prototypes used in this test were 3, 12 and 13.

Moreover, coils are powered at different frequencies. Table 5 shows a summary of the frequencies used in all tests. It also shows which coil is powered and with which frequency. We have tagged the frequency where the peak is registered with the symbol √. The lowest frequency value used to power the coils is 100 kHz. Due to the great variety of coils, the frequencies used to power them are different for each coil. However, we try to maintain some similar values for all the coils—100 kHz, 1000 kHz and 1500 kHz. The highest frequency used to power the prototype 1 is 4000 kHz. The prototype that was powered with the lowest frequency is prototype 11, whose maximum frequency was 1500 kHz. Frequencies used to power the coils are selected according to the points where coils present the mayor differences as a function of the salinity levels.

**Figure 5:**
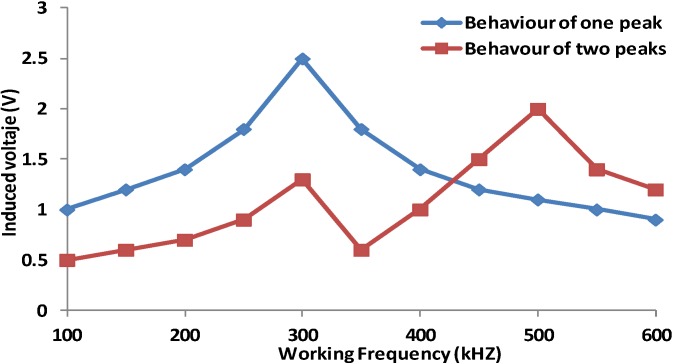
Example of possible behaviors of different prototypes.

The frequency where each prototype can be used as a salinity sensor is the frequency where the saline solution produces a different alteration of magnetic field and generates changes in the induced voltage. Generally, this frequency is the same that the peak frequency where the induced coil presents its highest voltage. In some cases, prototypes present just one peak frequency, but in other, prototypes present more than one peak frequency (frequencies where the induced voltage is higher than the induced voltages at lower and higher frequencies). Figure 5 shows these two possibilities representing the output voltages of the induced coil as a function of working frequency. When we have two or more peak frequencies, the highest one will be called maximum peak frequency.

**Table 5:** Summary of frequencies used for each prototype.

	Test 1				Test 2					Test 3					Test 4		Test 5	
Freq. (kHz)	P1	P2	P3	P4	P5	P6	P7	P8	P9	P5	P6	P7	P8	P9	P10	P11	P12	P13
**100**	x	x	x	x	x	x	x	x	x	x	x	x	x	x	x	x	x	x
**150**														x				
**200**														x		x		
**250**			x	x		x					x	x	x	x	x			
**280**														x				
**300**				x									x	x			x	x
**330**													x					
**350**													x	x				
**380**				x														
**400**								x				x	x	x		x		
**425**				√														
**450**												x	x			x		
**480**												√						
**500**	x	x	x	x	x	x	x			x	x	x	x		x	x	x	x
**550**												x				x		
**600**			x	x					√				x	x		√	x	
**620**																x		
**650**												x				x		
**700**			x	x		x			x		x		x			x	x	
**750**					x		x	x				x	x		x			
**800**			√	x		x		√			x		x	x		x	√	x
**850**												√						
**900**				x	√	x	√	x			x	x	x			x	x	√
**950**												x						
**1000**	x	x	x	x	x	x	x	x	x	√	x	x	x	x	x	x	x	x
**1100**					x	x	x	x			x	x	x	x				x
**1200**						√					√	x		√				
**1250**		x	x		x		x						x	x	x			
**1300**						x			x		x	x		x				x
**1400**						x					x							
**1500**	x	x	x	x	x	x	x	x	x	x	x	x	x	x	√	x	x	x
**1600**						x					x		√					
**1700**						x					x		x					
**1720**															x			
**1750**			x					x										
**1800**						x					x		x					
**1840**		√																
**1900**						x					x		x					
**2000**	x	x	x		√	x	x	x	x	x	x	x	x	x	x		x	x
**2250**		x								x					x			
**2500**	x	x			x	x	x	x	x	x	x	x	x	x	x			
**2600**										x								
**2700**										x								
**2750**	x	x													x			
**2800**										x				x				
**3000**	x	x			x	x	x	x	x	x	x	x	x	x	x			
**3500**	x																	
**3753**	√																	
**4000**	x																	

#### 4.1.1. First Test: Changes in the Number of Spires Maintaining the Spires Relationship

**Figure 6:**
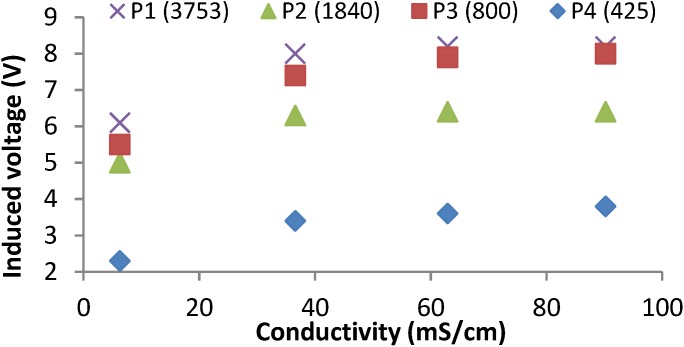
Induced voltages for best frequencies of prototypes from 1 to 4 at test 1.

In this test, we measured the induced magnetic field of the prototypes from 1 to 4 for four different water salinities. For prototype 1, the frequency peak is registered at 3753 kHz. For prototype 2, the frequency peak is registered at 1840 kHz. For Prototype 3 the frequency peak is registered at 800 kHz and, finally, for Prototype 4 the frequency peak is registered at 425 kHz. For these four prototypes, it is possible to distinguish the four different samples. Figure 6 shows the induced voltage of each prototype for each salinity sample. In all cases, there exists a positive correlation between the induced voltage and the salinity level. This correlation points that these prototypes can be used as salinity sensors. For all cases, when the salinity level increases, the induced voltage also increases.

#### 4.1.2. Second and Third Test: Change the Spires Relation

**Figure 7:**
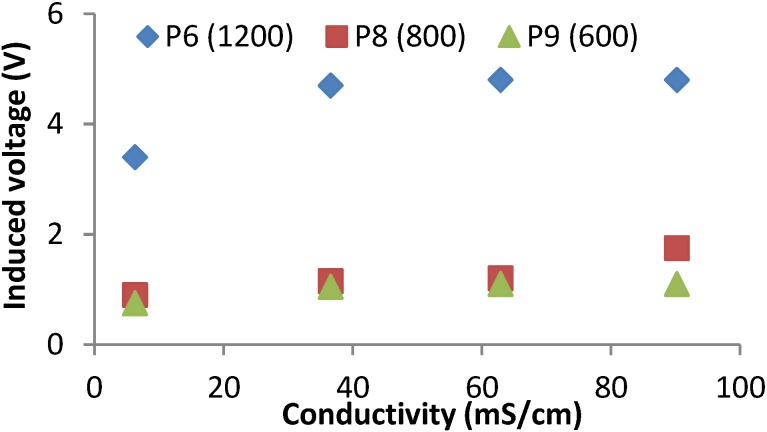
Induced voltages for best frequencies of prototypes from 5 to 9 at test 2.

In these two tests, we measured the induced magnetic field of prototypes from 5 to 9 at four different water salinities. Those prototypes are characteristics because one of the coils has 30 windings and the other one has variable windings (15, 30, 60, 90 and 120). During the second test we measured the induced field in the coils with 30 windings and powered the coils of different windings. Prototype 5 presents two frequency peaks, one at 900 kHz and other at 2000 kHz. In prototype 6 the frequency peak is registered at 1200 kHz. In prototype 7 the frequency peak is registered at 900 kHz. Prototype 8 also presents two peaks, one at 800 kHz and other at 1750 kHz. Finally, prototype 9 presents two peaks, one at 600 kHz and the second one at 1300 kHz. In this case, not all peak frequencies are useful to detect salinity variations. Only the prototypes 6, 8 and 9 at their peak frequencies offer results that correlate different voltage inductions at different salinities and this is shown in Figure 7. All tested prototypes used in test two offer very poor results. There is no clear linearity between data and induced voltage. On the other hand, as in the first test, in all cases the induced voltage increases as a function of the salinity level. Four of the five prototypes (5, 6, 8 and 9) present two different peaks in the working frequency.

**Figure 8:**
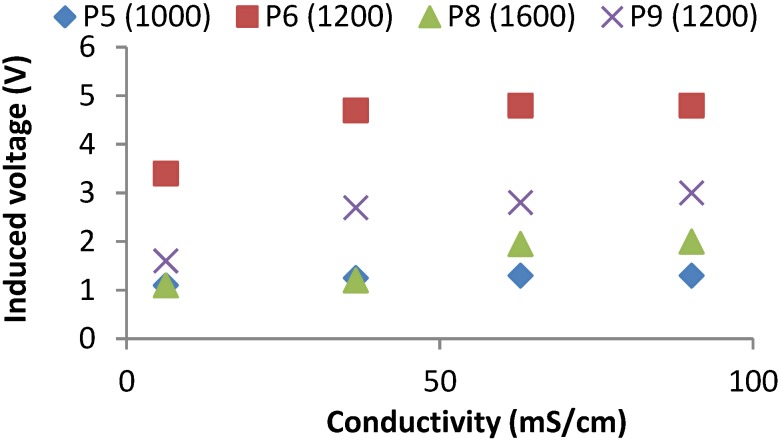
Induced voltages for best frequencies of prototypes from 5′ to 9′ at test 3.

During the third test, we used the prototypes 5′ to 9′ powering the coils with 30 windings and measuring the induced field in the coils of different windings. The frequency peaks for each prototype are the following; prototype 5′ presents two peaks, one at 1000 kHz and other at 2700 kHz; Prototype 6′ registers the peak at 1200 kHz; Prototype 7′ also presents two peaks, the first one is registered at 480 kHz and the second one appears at 850 kHz. Prototype 8′ presents four peaks at 330 kHz, 760 kHz, 1100 kHz and 1600 kHz. Finally, Prototype 9′ presents three peaks. The first one appears at 280 kHz, the second peak is at 1200 kHz and the last one is registered 2800 kHz. The induced voltages at the frequency where prototypes are able to detect changes in conductivity are represented in Figure 8. In this case, not all the frequency peaks are useful to detect salinity variations. Thus, prototype 7 is not useful. Four of the five prototypes (5, 7, 8 and 9) present more than one induction peak. Moreover, in all cases at the frequency peak, the induced voltage increases with the salinity of samples.

#### 4.1.3. Forth Test: Change the Wire Diameter

In these tests, we measured the induced magnetic field of prototypes 3, 10 and 11 using the same four different water samples. All prototypes used in this test have the same number of windings in both coils (20 windings in the powered coil and 40 in the induced coil). However, the diameter of copper wire used is different for each one. Prototype 3 is coiled with a copper wire of 0.4 mm. The diameter of copper wire for prototype 10 has 0.6 mm, and prototype 11 uses a copper wire of 0.8 mm. The frequency peaks for each prototype are the following: for prototype 3 it is registered at 1840 kHz, for prototype 10 the frequency peak appears at 1500 kHz and Prototype 11 presents the frequency peak at 600 kHz. Prototype 10 is not suitable to detect changes in salinity levels working at their frequency peak, but nevertheless prototype 11 can be used for this goal.

**Figure 9:**
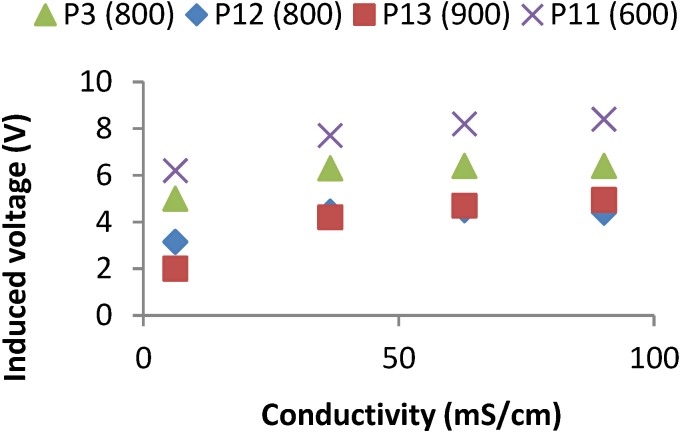
Induced voltages for best frequencies of prototypes 3, 11, 12 and 14 at tests 4 and 5.

In Figure 9, it is possible to see the relation between the salinity level and the induced voltage of prototype 11 at 600 kHz. In all cases, the induced voltage increases as a function of the salinity working at the frequency peak. The maximum voltages are higher than values in the fourth test. Prototype 11 raises an induced voltage of 8.4 V for the highest salinity sample. Moreover, all the prototypes present just one frequency peak.

#### 4.1.4. Fifth Test: Change the Coil Diameter

In these tests we measured the induced magnetic field of prototypes 3, 12 and 13 at four different water salinities. Prototypes used in this test have the same number of windings in both coils (20 in the powered coil and 40 in the induced coil). However, the diameter of coils is different for each one. Prototype 3 has a diameter of 25 mm, while the diameter of coils for prototype 12 is 15 mm and prototype 13 has 35 mm. The frequency peaks for each prototype are the following ones. For Prototype 3 the frequency peak is registered at 800 kHz, for prototype 12 the frequency peak is registered at 800 kHz, and finally, Prototype 13 registers the frequency peak at 900 kHz. In this case, all prototypes are able to distinguish between different salinities working at their frequency peaks. Results are shown in Figure 9. As we can see, in all cases the induced voltage increases depending on the salinity. Moreover, all these prototypes present only one frequency peak.

#### 4.1.5. Summary of Tests for Physical Characterization of the Sensor and Election of Prototype

As a summary of the performed tests, we can highlight the following facts:

- A total of 13 different prototypes were tested, four of them with two different powered/induced coil configurations.
- Those 17 combinations of coils were powered at frequencies from 100 kHz to 4000 kHz.
- Each combination has one or more peaks of induction at different frequencies
- Generally, peaks of induction represent the frequency where the prototypes are be able to detect conductivity changes.
- From 17 different configurations, 14 of them are able to detect conductivity changes.
- The frequency at which the prototypes are able to determine the conductivity is shown in Table 6.

In Figure 9, it is possible to see the relation between the salinity level and the induced voltage of prototype 11 at 600 kHz. In all cases, the induced voltage increases as a function of the salinity working at the frequency peak. The maximum voltages are higher than values in the fourth test. Prototype 11 raises an induced voltage of 8.4 V for the highest salinity sample. Moreover, all the prototypes present just one frequency peak.

To choose the prototype that will be used as a conductivity sensor, several factors must be considered. These factors are the frequency peak, voltage variation between saline solutions, and size of the prototype. It is desirable that the selected prototype presents its frequency peak at low frequencies, since the electric components for the final circuit are cheaper for low frequencies. Higher voltage variation between the saline solutions indicates higher sensibility of the sensor, which makes the monitoring process better. Finally, the coil size (number of windings and diameter) influences the magnitude of the generated magnetic field and it is desirable not to have big magnetic fields, to avoid the effect of boundaries. Moreover, smaller coils are cheaper to produce.

**Table 6:** Frequency of working for each prototype.

Prototype	Frequency (kHz)	Prototype	Frequency (kHz)	Prototype	Frequency (kHz)	Prototype	Frequency (kHz)
1	3753	6	1200	6′	1200	12	800
2	1840	8	800	8′	1600	13	900
3	800	9	600	9′	1200		
4	425	5′	1000	11	600		

From the 14 combinations that are able to determine the variation of conductivity, we have to select one of them. As said above, the frequency is very important, so the prototypes with frequency peaks above 1000 kHz were dismissed as a feasible option. The prototypes that accomplish the premise of low frequency peak are prototypes 3, 4, 8, 9, 5′, 11, 12 and 13.

Another important factor is the voltage difference between saline solutions, so the prototypes that have less than 2 V of difference between saline solution 1 and saline solution 4 are also dismissed as a possible option. The sensors that meet all the requirements are prototypes 4, 11 and 13. The last factor to take into account is its size. For this reason, Prototype 13 was also dismissed because of its high diameter and it does not present improvement enough over the prototypes with smaller diameters. Finally, Prototype 4 and Prototype 11 are compared in Table 7 from an economic point of view. Attending to the data presented in Table 7, the selected device is Prototype 4. It costs half as much as Prototype 11. Although this difference is less than a Euro, the number of devices we could need in a medium or large wireless sensor network could be very high, so in the end, the cost difference can be considerable. In some studies, there are more than 50 devices [30,32,33] and the sensors will be left different depths (supposed to be 10 for our calculations). Considering this amount of sensors, the budget using Prototype 4 instead of Prototype 11 is more than 200€ lower.

**Table 7:** Cost comparison of copper in each prototype.

Parameter	Prototype 4	Prototype 11
Coil diameter (mm)	25	25
Copper wire diameter (mm)	0.4	0.8
Spires in powered coil	40	20
Spires in induced coil	80	40
Volume of copper wire used (mm^3^)	1184	2369
Price of copper for Prototype (€)	0.42	0.85

### 4.2. Determination of Minimum Cell Volume

In this subsection, we are going to present the results of the tests performed to determine the minimum cell volume. First of all, we describe the concept of minimum cell volume, defined for the first time in our previous work [48]. Finally, the data of the performed tests are presented and analyzed.

As described before, there are two main methodologies to measure the conductivity, the inductive and the conductive one. In the conductive method, the volume of water is not related with the output voltage. It is only related with the amount of ions, area of copper electrodes and distance between copper electrodes. In inductive methodology the water volume is very important. This is because of the electromagnetic field produced by the powered coil extends beyond the space occupied by the coils. It is necessary to known the volume that has this electromagnetic field. The minimum cell volume is the minimum water volume necessary to cover all the extension of the magnetic field.

**Figure 10:**
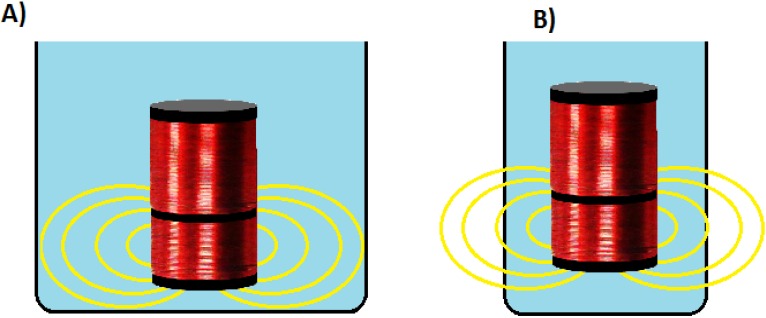
Example of containers of water that accomplish the minimum cell volume (**A**) and do not accomplish it (**B**).

This volume must be covered by water during the calibration and during the sensor operation. Otherwise, the induced voltage will be different generating wrong values of water conductivity. Figure 10 explains this situation with two examples. In (A) the volume of water is big enough to cover all the magnetic field represented in yellow lines. However in (B) the used container is too small and part of the magnetic field is outside the water. To find out that volume, the easiest way is using a simulator. Nevertheless, there is not any simulator that takes into account the attenuation effect of the electromagnetic waves into water with different conductivities using a coreless coil. Thus the only way is to perform the test described below.

**Figure 11:**
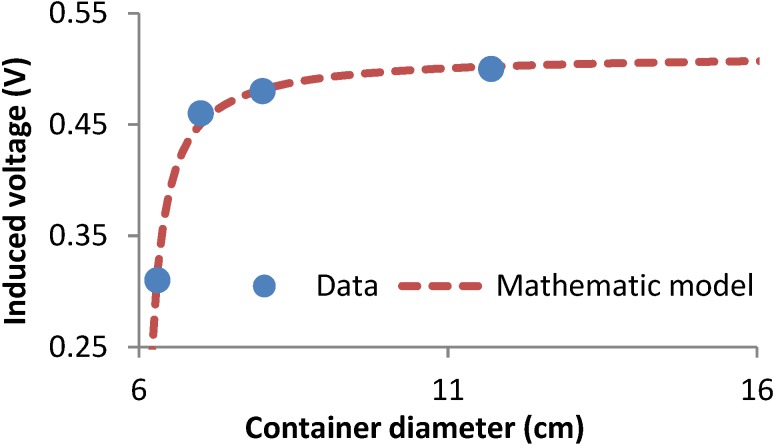
Results of the first test to find out the minimum cell volume.

The first test is aimed at finding the width of the electromagnetic field generated by the coil. Fixing the height of water and using different glass containers with different diameters, they are filled with the same water sample. The prototype is introduced inside the containers and the value of the induced voltage is recorded. These results can be seen in Figure 11, where the obtained data and the analytical model that models the data behavior are represented:

(7) $$Vout\;\left(V\right)=\frac{78.85-12.94\times D\left(cm\right)}{150.38-25.20\times D\left(cm\right)}$$

where *Vout* is the induced voltage (in volts) and *D* is the diameter of crystal container (in cm). The data corresponds to the output voltage of the induced coil when it is introduced inside the containers with different diameter (from 6.3 cm to 11.7 cm). From Equation (7), it is possible to know that the minimum diameter is 25 cm. From this size, when we increase the diameter, the output voltage does not change (at level of second decimal).

**Figure 12:**
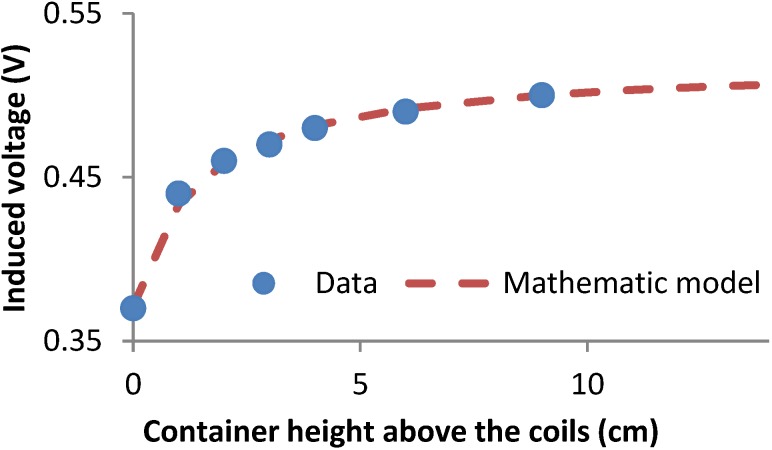
Results of the second test to find out the minimum cell volume.

The second test performed is aimed to find the height of the electromagnetic field generated by the coil. For this test, the container of 11.7 cm of diameter is used and the height of water was increased after each measure. We started with a height of water that only covers the coils; we call it as 0 cm. Then the level of water is increased with intervals of 1 cm after each measure. The output voltage at each height of water is related with this water level and it is shown in Figure 12. The obtained data and the mathematical model (Equation (8)) are shown in Figure 12:

(8) $$Vout\;\left(V\right)=\frac{0.52+0.52\times H\left(cm\right)}{1.40+H\left(cm\right)}$$

where *Vout* is the induced voltage (in Volts) and *H* is the height of water that covers the coil (in cm). From the analytical model, it is possible to extract the height above the coils for the minimum cell volume of 20 cm. At 20 cm above and below the coil, if the height of water increases, the output voltage does not change (at the level of the second decimal). Considering the height of the coils (8 cm), the height above and below the coils is 20 cm, and the height of the magnetic field extension is 48 cm, then the minimum cell volume is 23.5 L.

### 4.3. Calibration

Once the minimum cell volume is known, the calibration can be performed. Nevertheless the volume necessary to meet the minimum cell volume requirements are too high to easily operate under laboratory conditions. Instead of using a container with 48 cm height and 25 cm diameter, a container with 28 cm height and 11.7 cm diameter is used. Thanks to our analytical models, it is possible to calculate the compensated output voltage due to the containers volume used, which is lower than the minimum cell volume. The compensation rate is 3.2%. By using this value is possible to correct the output voltages obtained in this test.

**Figure 13:**
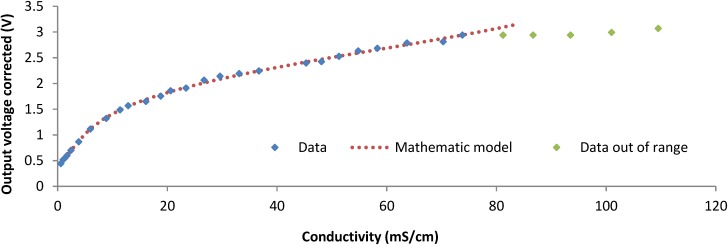
Representation of data of calibration process.

The calibration process is done with more than 30 samples, starting with the lowest conductivity value (0.585 mS/cm) and adding NaCl in small quantities after each measure to increase the salinity level. The value of conductivity is measured after adding NaCl with a CM 35+ conductivity meter. The highest value of conductivity tested is 109.5 mS/cm. At each conductivity level, the output voltage of the salinity sensor is recorded. The values of output voltage obtained with the induced coil are corrected by applying a correction factor. When values are corrected, we can see that at 73.8 mS/cm the output voltage is 2.94 V. When the conductivity value increases up to 86.7 mS/cm, the output voltage does not change. After 86.7 mS/cm the output voltage starts to increase again but irregularly, so 73.8 mS/cm is defined as the last point of our measurable range. Then, the measurable range of our sensor ranges from 0.585 mS/cm to 73.8 mS/cm. However, it is expected that the sensor will be able also to work at lower values. The obtained data are presented In Figure 13.

It is divided into two groups; the group Data represents the values that are in the working range; Data out of range presents those values that are out of the working range of the sensor, and Mathematic model is the analytical model that predicts the behavior of our sensor in the working range. The analytical model shown in Equation (9) is obtained using mathematical software (Eureqa [58]). The analytical model has a correlation coefficient R^2^ of 0.9985 and a mean absolute error of 0.84. In Equation (9) we can see the conductivity (*Cond*.) and its relationship with the induced voltage (IV). The equipment used to measure the output voltage has an acceptable accuracy (0.01 V) compared to the collected values:

(9) $$Cond.\;(\frac{mS}{cm})=-0.83\times IV^{5}(V)+3.96\times IV^{4}(V)-9.41\times IV^{2}(V)+16\times IV\;(V)-4.93$$

Once the mathematical model is obtained, it is time to verify our model. In order to do it, we used five different saline solutions of unknown conductivity. Even though we do not know the conductivity value of the solution, those values are inside the range of the mathematical model. Those solutions are measured with our prototype and the obtained induced voltages are converted into conductivity values in mS/cm using Equation (9). Then, the solutions are measured with the commercial conductivity sensor (CM35+) and we compared the lecture of the commercial device (real value) with our predicted value (equation value). The results are shown in Table 8. The absolute error and the relative error of those measures are calculated in order to have some information about the accuracy of our prototype and the mathematical model developed by us. The mean relative error is 2% and the maximum 8%, the mean absolute error is 0.88 mS/cm and the maximum 2.03 mS/cm. Those values indicate that our prototype has good accuracy for monitoring the changes of conductivity. The sensibility of our prototype, that is, the minimum variation of conductivity that the prototype is able to detect, is determined by the minimum variation of voltage that we are able to detect. This variation is 0.01 V. It is possible to determine the sensibility for different ranges as can be seen in Table 9.

**Table 8:** Verifying measures and the relative and absolute error.

Real Value (mS/cm)	IV (V)	Equation Value (mS/cm)	Relative Error (%)	Absolute Error (mS/cm)
1.72	0.59856	1.72	0%	0.00
11.38	1.48608	11.37	0%	−0.01
26.7	2.064	28.73	−8%	2.03
45.3	2.3994	44.37	2%	−0.93
58.3	2.6832	59.74	−2%	1.44

**Table 9:** Sensibility of our prototype with the mathematical model (3) at different ranges.

Sensibility (mS/cm)	From (mS/cm)	To (mS/cm)
0.1	0.6	5.5
0.2	5.5	11.5
0.3	11.5	18
0.4	18.1	28
0.5	28.1	41
0.6	41.1	86.7

## 5. Conclusions

For the correct management of water resources, continuous monitoring of water parameters is required. In groundwater resources, salinization is a serious problem. Several authors have tried to perform continuous measurements of this parameter. The difficulty of continuous monitoring can be solved using an appropriate wireless sensor network in order to gather the measurements on site when required, but many difficulties arise, so it is necessary to design suitable, simple and inexpensive sensors. This paper has presented the development and test of a specific sensor for monitoring the groundwater salinization process. Several prototypes are tested in order to find the best electrical coils combination, for obtaining the best correlation between induced voltage and the electric conductivity of water samples. The first test has been performed to choose the best sensors in terms of the size of sensor, cost, maximum induced voltage and frequency peak. The selected prototype is tested to find out its minimum cell volume. The sensor has also undergone a calibration process with more than 30 samples, getting thus a mathematical equation that correlates the sensor signal (induced voltage) with the electric conductivity of water. The useful working range is comprised from 0.58 mS/cm to 73 mS/cm. Because of our initial aim were to measure conductivities from less than 1 mS/cm up to 58 mS/cm, we can trust this prototype.

This sensor can be used to design automatic processes like the ones described in different works such as [16,59]. In [59], the authors proposed an optimal pumping strategy, specific for a coastal aquifer, using simulations. This system needs real-time information about the salinity level in aquifers. Our developed sensor could be a solution for this system. In [16] the authors proposed a methodology for risk assessment of salinization process in aquifers based on simulations. Again, this simulation needs continuous information of real salinity level in aquifers.

As future work, we will focus our effort on providing a more precise definition of other parameters that can also affect the aquifers salinization, with the purpose of adding them to real time monitoring systems [60]. We also want to check the strength of our prototypes to the effect of corrosion and how this fact could affect the measurement results. We will carry out a study to evaluate what is the phase shift between the two coils of different prototypes at different frequencies and salinities.
